# A Rare Presentation of Maydl's Hernia

**DOI:** 10.1155/2014/184873

**Published:** 2014-11-18

**Authors:** Elroy Patrick Weledji, Martin Mokake, Marcelin Ngowe Ngowe

**Affiliations:** Department of Surgery, Faculty of Health Sciences, University of Buea, P.O. Box 126, Limbe, Southwest Region, Cameroon

## Abstract

We present a case of an unsual type of obstructed indirect inguinal hernia with impending strangulation. The operative findings revealed a sliding Maydl's hernia with an ischemic inner ileal loop and an adherent inflamed appendix. This case highlights the importance of intraoperative examination of the intra-abdominal bowel loops proximal to the hernia sac of an incarcerated, obstructed, or strangulated hernia.

## 1. Introduction

Maydl's hernia is a rare type of incarcerated hernia popularly known as a hernia in “W” which describes the orientation of the bowel in the hernia sac and the vulnerability of the central segment of bowel to undergo intra-abdominal closed-loop strangulation which may go unnoticed. This is an unusual case involving the appendix although it was not classically in the hernia sac to suggest a coexistent Amyand's hernia.

## 2. Case Presentation

**Figure 1:**
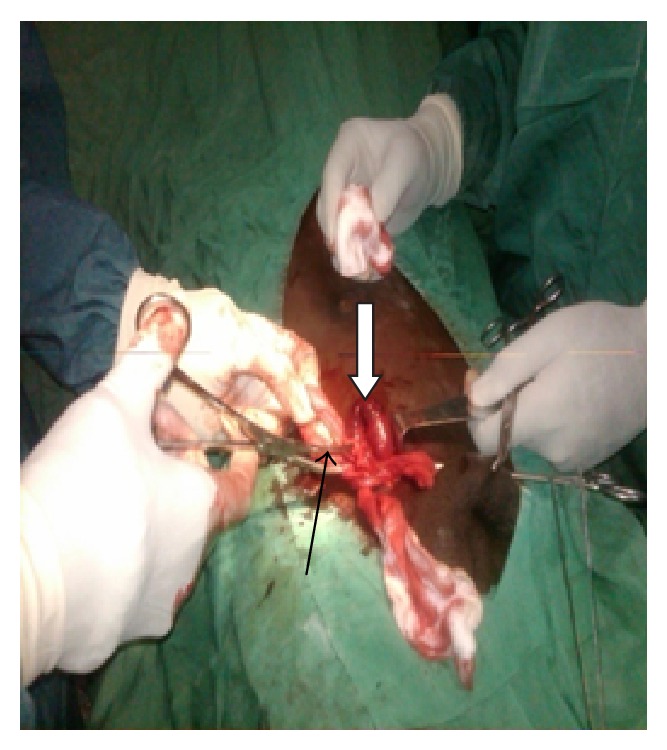
Loop of ileum with ischemia at convex margin (*white arrow*) and adherent inflamed appendix (*black arrow*).

A 30-year-old man was admitted as an emergency with a 24 hr history of a sudden onset painful irreducible right inguinal swelling following a meal. The groin swelling had been present for over a year associated with intermittent pain but had suddenly become bigger. The pain at presentation was associated with vomiting, progressive abdominal bloatedness, and constipation. On examination he was distressed with a BP 110/40 mmHg, pulse 88/min, respiratory rate 28/min, and a temperature of 37.5°C. There was a tender, tense, and irreducible groin swelling of ~5 cm in diameter. The diagnosis of a strangulated inguinal hernia was made. He underwent an emergency inguinal exploration following a rapid resuscitation with intravenous fluids and analgesia. A right oblique groin incision revealed an indirect hernia sac with some free fluid. The tight deep inguinal ring was widened and this revealed an intra-abdominal aperistaltic intervening loop of ileum which was ischemic at its convex margin (Figure 1). There was an adherent inflamed appendix which may have been reactionary, adjacent to the hernia sac contents. The ischemic loop was covered with hot moist gauze for 10 minutes until the normal lustre and peristaltic wave returned. The appendix was freed from the hernia sac and excised (Figure 1). The hernia sac was transfixed and reduced and a modified Bassini repair of the hernia was performed. This consisted of a tension-free apposition of the inguinal ligament to the conjoint tendon associated with plication of the transversalis fascia up to the deep ring using 2.0 nylon. He made good recovery and was discharged on the fifth postoperative day.

## 3. Discussion

**Figure 2:**
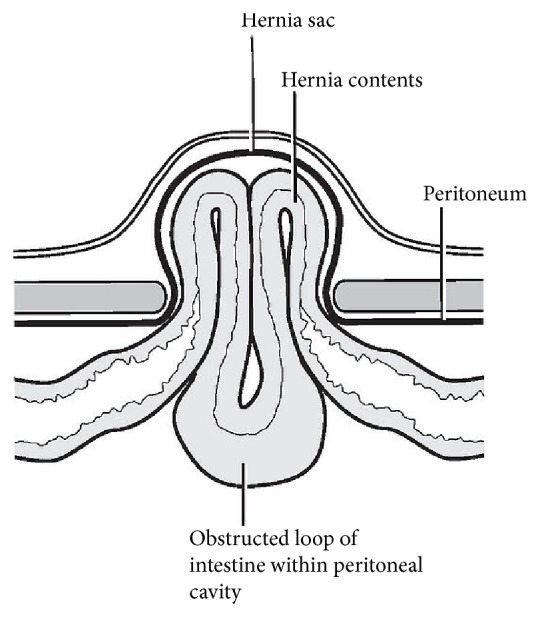
Schematic diagram of Maydl's hernia (strangulated intestine at apex of “W”).

Maydl's hernia is a double loop hernia with the middle, internal loop strangulated (Figures 1 and 2) [1]. It is a rare variety (<2%) of strangulated inguinal hernia first described by the Austrian surgeon Karel Maydl in 1895 [2]. When two adjacent loops of bowel are in the sac, the intervening portion in the abdomen is the first to suffer if the neck of the sac is tight, because it is the centre of the whole loop involved. Thus, the strangulated piece which is at the apex of the “W” is intra-abdominal and can be missed at surgery from a fatal misjudgment in observing two viable loops in the hernia sac [1–3] Further examination of intra-abdominal bowel proximal to the neck of the sac is important. Long-standing hernias may predispose to more bowels being dragged into the sac. Adhesions would predispose to the “W” configuration permitting more mobile loops to herniate further into the sac including the appendix as in this case [1–4]. Most of the reported cases are in Africa and are attributed to the high incidence of untreated hernias [4].

The association with Amyand's hernia is even rarer. It is of historical interest that in 1735 Claudius Amyand removed the appendix of an 11-year-old boy through a groin incision for a scrotal hernia, arguably the first appendicectomy to be performed although without the intention of curing appendicitis [5]. In Amyand's hernia the appendix may be normal, inflamed, or perforated [6]. A scrotal presentation can be mistaken for an acute hydrocoele, testicular torsion, or epididymoorchitis [5, 6]. Preoperative diagnosis is uncommon because imaging is not a routine in inguinal hernias. The surgical management is based on the Losanoff and Basson classification [7]. A type 1 Amyand hernia has a normal appendix. A hernia reduction and mesh repair without appendicectomy is done. For type 2 with acute appendicitis but no abdominal sepsis, an appendicectomy is performed through the hernia and also a primary repair with no mesh. Type 3 with acute appendicitis and abdominal wall or peritoneal sepsis requires a laparotomy, appendicectomy, and primary repair of hernia. For type 4, where acute appendicitis is associated with a related or unrelated pathology, the management is as for types 2 and 3 hernias but the second pathology should be investigated and treated appropriately. Left-sided Amyand's hernia is very rare and may be associated with situs inversus, malrotation, a mobile caecum, and an excessively long appendix [8]. Appendicectomies for left-sided hernias should be a routine irrespective of being inflamed or not. This would avoid future doubt if appendicitis occurred [9]. The use of prosthetic mesh in the repair is normally contraindicated in inflammation and infection [6, 8]. In this case, where there was no strangulation nor resection anastomosis of the gut, a mesh hernioplasty would have been most appropriate.

## 4. Conclusions

Maydl's hernia although rare should be suspected in patients with large incarcerated hernia, with evidence of strangulation or peritonitis, or with viable loops of intestine in the hernia sac. Examination of the bowel loops proximal to the obstructing hernia ring is vital to avoid return of nonviable bowel to the abdomen during repair. In Amyand's hernia the decision to perform an appendicectomy and type of repair depends on the clinical presentation and is guided by Losanoff and Basson's criteria.
